# Providing Stability to High Internal Phase Emulsion Gels Using Brewery Industry By-Products as Stabilizers

**DOI:** 10.3390/gels7040245

**Published:** 2021-12-01

**Authors:** Adrián López-García, Gemma Moraga, Isabel Hernando, Amparo Quiles

**Affiliations:** Departamento de Tecnología de Alimentos, Universitat Politècnica de València, 46022 Valencia, Spain; adlogar2@etsii.upv.es (A.L.-G.); gemmoba1@tal.upv.es (G.M.); mquichu@tal.upv.es (A.Q.)

**Keywords:** oil structuring, physical stability, stabilizers, structure, biopolymers, rheology, cryo-SEM, vegetable oils

## Abstract

The modern brewing industry generates high amounts of solid wastes containing biopolymers—proteins and polysaccharides—with interesting technological and functional properties. The novelty of this study was to use raw by-product from the brewing industry in the development of high internal phase emulsion (HIPE) gels. Thus, the influence of the emulsion’s aqueous phase pH and the by-product’s concentration on structural and physical stability of the emulsions was studied. The microstructure was analyzed using cryo-field emission scanning electron microscopy. To evaluate the rheological behavior, oscillatory tests (amplitude and frequency) and flow curves were conducted. Moreover, the physical stability of the emulsions and the color were also studied. The increase in by-product concentration and the pH of the aqueous phase allowed development of HIPE gels with homogeneously distributed oil droplets of regular size and polyhedral structure. The data from the rheology tests showed a more stable structure at higher pH and higher by-product concentration. This study widens the possibilities of valorizing the brewing industry’s by-products as stabilizers when designing emulsions.

## 1. Introduction

Food and by-product management is one of the main challenges that the agro-industrial sector faces in the 21st century. Modern brewing is an industry producing high amounts of by-products, including solid and liquids. Throughout the process, diverse solid wastes are produced, mainly spent grains, trub, and spent yeast [1].

Trub is an effluent mainly composed of hop particles, colloidal proteins, and residual beer liquor. Spent yeast comprises excess yeast recovered from sedimentation in the tank. Trub and spent yeast generation can reach 0.4 and 3 kg per hL of beer, respectively [2]. Valorizing by-products is a promising strategy to reduce the wastes and to produce new added value ingredients or components [3]. To date, these beer by-products are mostly used as animal feed [4]. However, there is a trend trying to valorize them, such as their use in food supplements and functional foods [5], as a stored food insect protector [6], as antioxidant and antimicrobial [7] and to be used as free fat protein [8].

Trub is a source of fiber and protein, obtained from spent hops and the wort cooking, where proteins denature [7]. Spent yeast is also a source of lignocellulosic components and rich in proteins [9]. The emulsifying properties of spent yeasts have already been investigated and are attributed to β-d-glucans and mannoproteins [10]. Authors have evaluated the potential use of isolated mannoproteins and glucans from spent yeast as a fat replacer in mayonnaise and salad dressings as well as a carrier for microencapsulation [11,12,13,14]. Saraiva et al. [11] isolated protein from trub to produce high protein ice cream, Silva Araujo et al. [12] extracted β-glucan and mannoproteins from brewer’s yeast to replace xanthan gum in mayonnaise, de-Melo et al. [13] used mannoproteins from discarded brewer’s yeast in the formulation of a salad dressing to substitute syntethics agents, Paramera et al. [14] summarized the possibilities of using yeast cells for microencapsulation and Worrasinchai et al. [15] applied β-glucans from spent brewer’s yeast as a fat replacer in low calorie mayonnaises. However, none have evaluated the possibility of directly using beer by-products, without isolating carbohydrate or protein compounds, to produce high internal phase emulsion (HIPE) gels. HIPEs are a type of emulsion in which the volume of internal phase exceeds the close packing limit (φ > 0.74) inducing conformational changes in droplets shape due to the relatively low Laplace pressure [16]. These conformational changes produce “solid-like” systems exhibiting viscoelastic properties, which allow the structuring of non-plastic fats, such as vegetable oils, using a direct approach [17]. Because of the high degree of packing, these emulsions are highly resistant to creaming or sedimentation [16]; furthermore, the high degree of packing caused by the elevated internal volume fraction allows these systems to encapsulate, protect, and deliver bioactive compounds in high concentrations [18,19,20]. Moreover, concentrated emulsions may mimic the functionality of saturated plastic fats [21]. Replacing saturated fat in food products is challenging as this type of fat shows good oxidative stability and solid lipid functionality. In this context, emulsion gels have been used to fully replace margarine in cakes obtaining comparable functionalities and sensorial properties than non-replaced cakes [22].

Harnessing plant based agro-industrial by-products as sustainable raw materials is very attractive, but it is time and energy-consuming because of the need to purify the desired compounds [23,24,25,26]. Herein, we worked with a mixture of trub and spent yeasts without modification, except for drying. To the best of our knowledge, there is no evidence of non-modified by-products used to produce HIPEs. However, using by-products to stabilize emulsions is a potential area of research and, recently, it has been studied to produce other type of emulsions, such as Pickering emulsions [27].

This study aimed to structure sunflower oil to obtain HIPE gels using brewing industry by-products as stabilizers. This study focused on understanding the effect of the pH of the aqueous phase and by-product’s concentration on the emulsions’ structural and physical stability.

## 2. Results and Discussion

### 2.1. Powder’s Characterization

The water content of the powder was 3.70 ± 0.07% and over half the particles (53%) had a particle size between 90 and 106 μm, 42% between 63 and 90 μm, and 5% between 45 and 63 μm. Color attributes were L* = 71.78 ± 0.34, C* = 32.25 ± 0.63 and h* = 85.11 ± 1.68.

### 2.2. Microstructure

**Figure 1:**
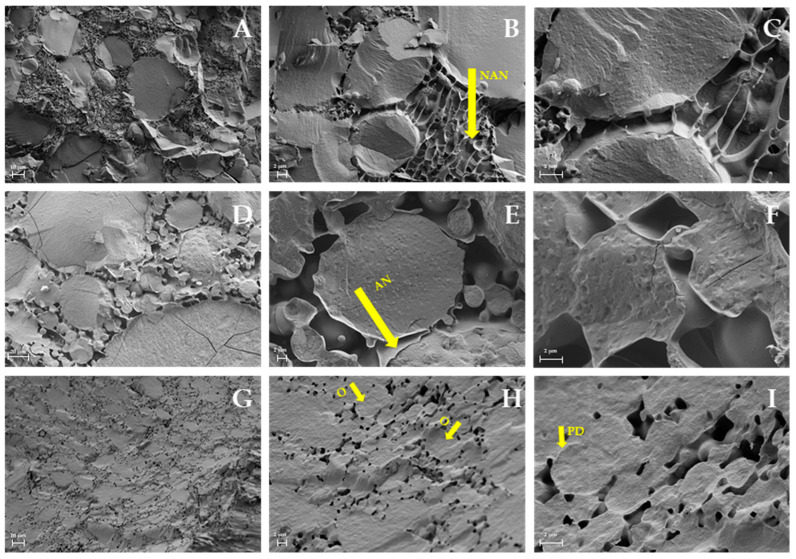
Microstructure of the emulsions observed using Cryo-FESEM. (**A**–**C**): 9H sample; (**D**–**F**): 12L sample; (**G**–**I**): 12H sample. (**A**,**D**,**G**): 500× magnification; (**B**,**E**,**H**): 2000× magnification; (**C**,**F**,**I**): 5000× magnification. Arrow NAN: non-adsorbed polymeric network; arrow AN: adsorbed polymeric network; arrow O: oil droplet; arrow PD: more polyhedric shape. (**A**,**G**) scale bar 10 µm; (**B**,**C**,**E**,**F**,**H**,**I**) scale bar 2 µm; (**D**) scale bar 20 µm.

Samples 9H, 12L, and 12H (as examples of limit values of pH—9, the lowest and 12, the highest—and concentration—L, the lowest and H, the highest) were observed using the Cryo-FESEM technique to study the effect of pH and by-product concentration in the microstructure of the emulsions (Figure 1). The effect of pH was studied comparing samples 9H and 12H. In the 9H emulsion (Figure 1A–C), oil droplets were mostly big and embedded in a continuous matrix, mostly constituted of the by-product components. In this emulsion, oil droplet distribution and size were not homogenous. The continuous phase was likely generated through weak interactions between the biopolymers constituting the by-product. Sample 12H (Figure 1G–I) had a smaller oil droplet size than 9H, and a droplet deformation, from spherical to polygonal, was observed. The surface of the oil droplets had a greater coating when pH increased, as observed when comparing Figure 1A–G. In sample 9H, a non-adsorbed polymeric network was observed that did not occur in sample 12H. This might produce destabilization because of a weak interaction between polymers, producing bridging flocculation as shown in Figure 1C, and thus indicate these by-products act as stabilizers when pH is increased.

To understand the influence of the by-product’s concentration, samples 12L (Figure 1D–F) and 12H (Figure 1G–I) were compared. A heterogenous oil droplet size was observed in sample 12L; oil droplets were connected and separated by an interphase made by the interactions between the biopolymers from the by-product. The 12H emulsion showed smaller, more polyhedric shape, and more packed oil droplets than 12L with more structuring. It seems that the stabilizer effect of the biopolymers from the trub is higher when the concentration increases, and the pH is high. Increasing the amount of stabilizer usually increases HIPE gels stability until a limit of concentration, where an excess can be self-defeating [28]. Further research would be required to optimize the fabrication process of the end product, including gel dispersion within the food matrix.

### 2.3. Rheological Behavior

**Figure 2:**
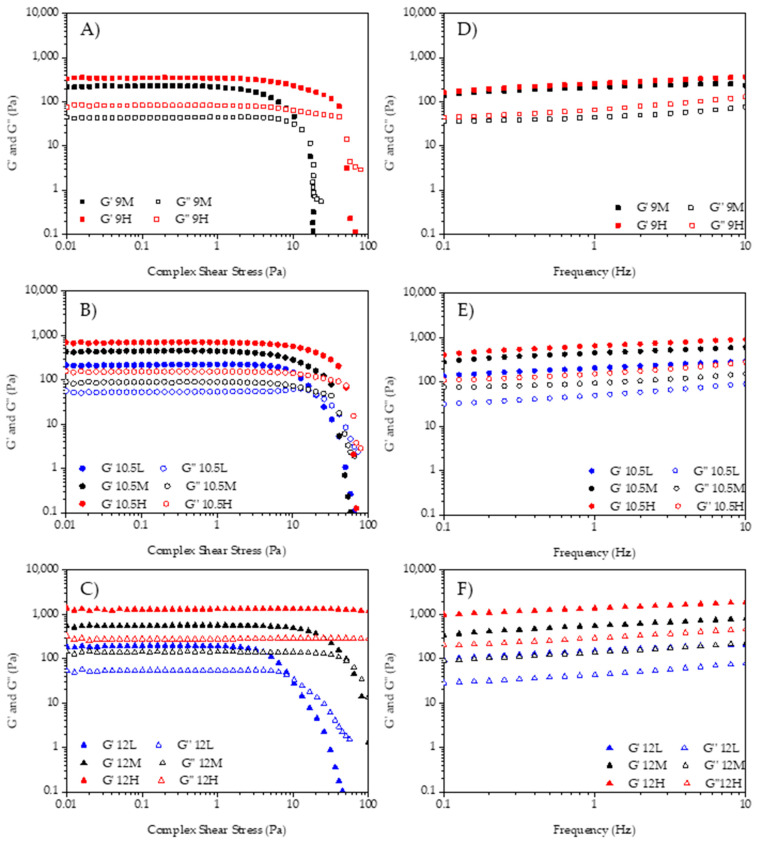
Rheological behavior of the emulsions. Left: amplitude sweeps; Right: frequency sweeps. (**A**,**D**) 9M and 9H; (**B**,**E**) 10.5L, 10.5M, and 10.5H; (**C**,**F**) 12L, 12M, and 12H.

The rheological properties were analyzed to understand the influence of the pH of the aqueous phase and the by-product concentration on the emulsion stability. Amplitude sweeps gave the linear viscoelastic region (LVR) of the emulsions. The extension of the LVR of the different samples at 1 Hz is shown in Figure 2, where values of the elastic modulus (G′) and the viscous modulus (G″) versus the shear stress wave amplitude are plotted. All samples had a higher G′ than G″ at lower amplitudes, meaning the emulsions had a solid-like behavior. The sample with the lowest pH and by-product concentration (9L) was not characterized due to its instability.

The crossover point, where G′ = G″, appeared at amplitudes closer or higher than 10 Pa in all samples. This characteristic point—where the transition from solid-like to liquid-like behavior occurs—moved to higher stress values when pH and concentration were increased. This shift of the crossover to higher shear stress meant that at higher pH and concentration more stable HIPE gels were obtained, as higher stress values were needed to break down the internal structure. Figure 2A shows that G′ of 9H was higher than G′ of 9M along the test and the crossover point’s shear stress increased because of the by-products increased concentration. In Figure 2B, G′ showed the same behavior as Figure 2A and showed higher values when the concentration increased (10.5H > 10.5M > 10.5L).

The crossover points of L, M, and H samples in Figure 2B showed less difference between them than pH 9M and H samples, but the G′ at these points was always higher as commented before.

Figure 2C shows the amplitude sweeps of samples 12L, 12M, and 12H. G′ increased as the concentration of the by-product increased, as occurred in the other samples (12H > 12M > 12L). The crossover point (flow point) showed greater stress differences between concentrations, as occurred in the pH 9 samples when by-product concentration was higher; however, sample 12H did not flow nor break its structure.

Figure 2D–F show the mechanical spectra, or the relationship between the viscoelastic modulus as a function of the frequency at a constant stress of 1 Pa (within LVR). In all the emulsions, G′ was higher than G″ along the entire range of frequencies studied and there was a low dependence between G′ and G″ and the frequency. This indicates tightly packed systems with a solid internal gel structure where these parameters are governed by the bulk and the interfacial properties. When comparing the effect of the concentration in terms of structural stability, it is shown how increasing the amount of by-product increases the values of G′ and G″. Thus, brewery wastes can be used as viable biopolymers to design concentrated emulsion.

For all the rheological parameters studied, there were significant interactions (*p <* 0.05) between the two factors by-product concentration and pH of the aqueous phase. The values of the viscoelastic modulus (G′, G″) at 1 Hz and 1 Pa are shown in Table 1.

When studying the effect of pH of the aqueous phase on G′, no significant differences (*p >* 0.05) were found among samples with low concentration (10.5L and 12L). Among medium concentration samples, only 9M (low pH) differed from 10.5M and 12M. However, among high concentration samples, there were significant differences, increasing G′ when pH was increased (9H < 10.5H < 12H). Thus, the higher the pH the more “solid-like” behavior. When comparing the influence of by-product’s concentration, no significant differences (*p >* 0.05) were found between 9M and 9H, but differences were found among samples with pH 10.5 and 12. Elastic shear modulus increased significantly (*p <* 0.05) as by-product’s concentration increased.

Regarding the viscous shear modulus (G″), when comparing the influence of the pH of the aqueous phase, no significant differences (*p >* 0.05) were found between the samples with the lowest concentration of by-product (10.5L and 12L), but differences were found for samples with medium and high concentration. In these samples, G″ significantly (*p <* 0.05) increased when increasing the pH of the aqueous phase. The by-product’s concentration does not significantly influence (*p >* 0.05) the samples with the lowest pH (9M and 9H) but had a significant effect (*p <* 0.05) on the samples with medium and high pH. In these samples, the viscous shear modulus significantly increased (*p <* 0.05) as the by-product’s concentration increased. Therefore, viscoelastic parameters (G′ and G″) increased their values when increasing the pH of the aqueous phase and by-product concentration. A local G″-maximum appears in Figure 2B on sample 10.5L at 20 Pa. This issue could be related to different factors such as internal friction due to the loss of stability. In general, the values of G′ and G″ are within the range of emulsions stabilized with plant fibers from by-products (10–1000 Pa [29] and 35–200 Pa [30]). Further, in the work by Huc-Mathis et al. [31], they achieved G′ and G″ values of 225 and 30 Pa, respectively. This indicates that samples with high concentration and high pH are strong emulsions formulated with by-products.

**Figure 3:**
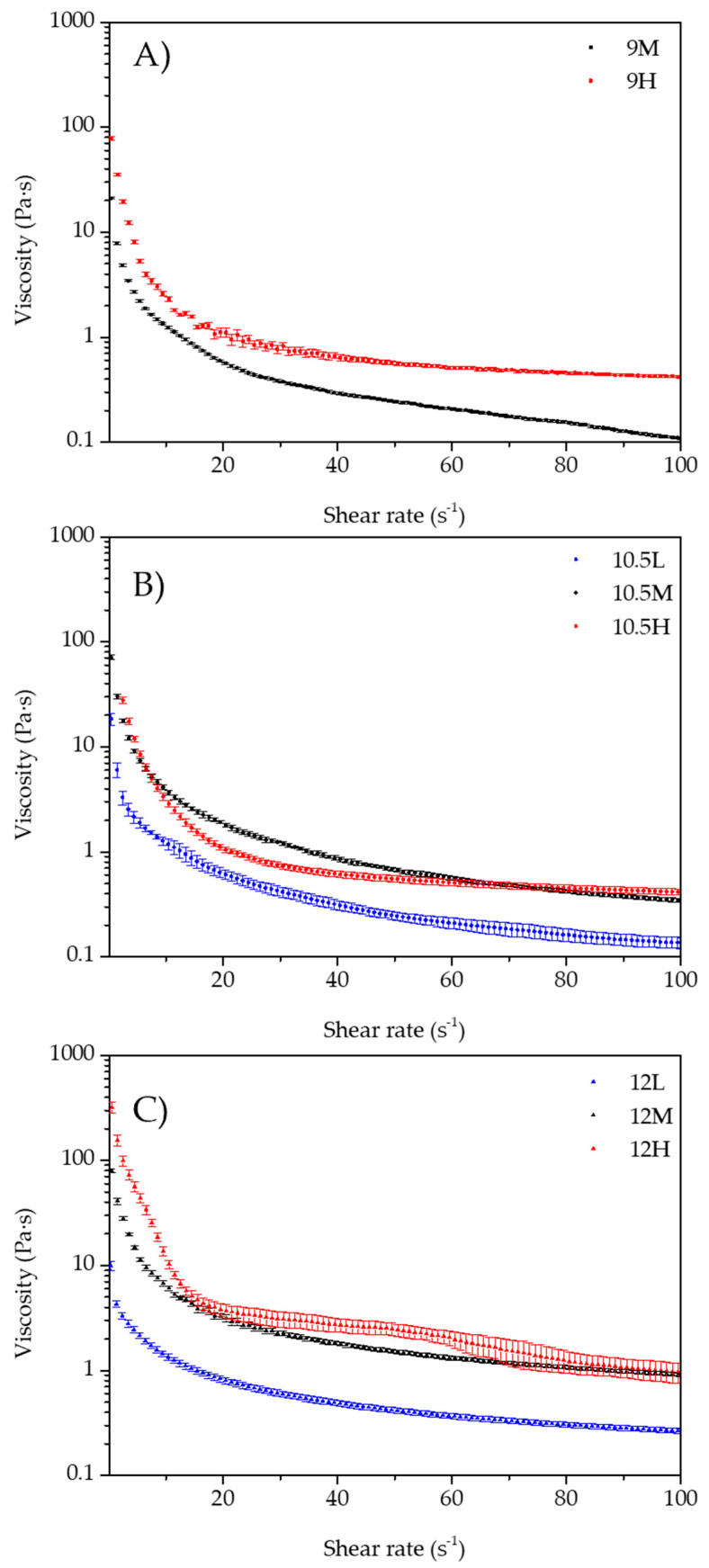
Flow curves of the emulsions. (**A**) 9M and 9H; (**B**) 10.5L, 10.5M, and 10.5H; (**C**) 12L, 12M, and 12H.

Figure 3 shows the flow curves of the different samples. All samples had a shear-thinning behavior (i.e., lower viscosity at higher shear rate).

All samples had similar flow curves, characterized by a rapid decline in the viscosity value at small shear rates and then reaching a stationary value (where the decay was very slow); furthermore, higher viscosities were observed at higher by-product’s concentration. Only sample 10.5H displayed an “anomaly” to this trend, because it showed a lower viscosity value than 10.5M at less than 50 s^−1^. After this point, viscosity was like 10.5M until at the end of the test when its value was slightly higher than 10.5M. The drop of viscosity is caused by the breakdown of the structure when exposed at high shear rates.

The values of viscosity at 50 s^−1^ are shown in Table 1 and shows the effect of pH of the aqueous phase was significant (*p <* 0.05) among samples. Furthermore, viscosity values increased when increasing pH; however, the samples 9H and 10.5H showed no significant differences (*p >* 0.05). The influence of the by-product’s concentration was significant for every pH, i.e., for samples of pH 9, 10.5, and 12 significantly higher viscosity values (*p <* 0.05) were observed as the by-product concentration increased. However, as shown in Figure 3B, samples with pH 10.5 presented an “anomaly” as 10.5H had a viscosity value at 50 s^−1^ significantly (*p <* 0.05) lower than 10.5M.

These results are related to the microstructure observations, where emulsions with smaller and more packed oil droplets were observed when increasing pH and the by-product concentration. Based on Liu et al. [19], this can be linked to the increase in viscosity due to three reasons: (1) the smaller the droplets, the higher the number of interactions per unit of volume; (2) reducing droplets’ volume diminishes their deformability because of the increase in Laplace’s pressure; and (3) increasing the by-product concentration could cause increased interactions between droplets due to a higher level of flocculation.

Results can also be explained by the effect of increasing the pH of the aqueous phase on the trub components. Spent brewer’s yeast has a content of 40% crude protein, 59% carbohydrates and 1% lipids. Its cell wall is strong, thick, and resistant and has a variable composition, but it is mostly made from β-1,3/β-1,6 glucans and glycoproteins [32]. Increasing the pH of the aqueous phase up to 12 probably solubilized proteins and some polysaccharides from the cell wall and degraded the glucose chains from the insoluble compounds [33]. Therefore, different biopolymers from the cell wall can be released to the aqueous phase. The release of glucans to an alkali medium could degrade its structure with a reduction in branching and the break of covalent bonds between mannoproteins and β-1,3/β-1,6 glucan chains, which decreases de degree of polymerization [32]. Li and Karboune [34] studied the functional properties of mannoproteins from spent brewer’s yeast and found an increase in its solubility when increasing pH from 3 to 9. We hypothesize that at higher pH the solubility of mannoproteins increases. Moreover, alkaline treatment cleaves the glycosylphosphatidylinositol anchor between mannoproteins and β-glucan [32], and therefore, β-glucans remains insoluble and increase the viscosity of the aqueous phase [33]. Moreover, by adding more by-product, the viscosity of the bulk phase was enhanced. This is likely due to the higher amount of yeast cell debris (an insoluble fraction of the by-product, made from large proteins and polysaccharides) produced during preparing the aqueous phase that improved the stability and also due to the solubilization of proteins which can act as thickeners and emulsifiers [35].

### 2.4. Physical Stability

**Figure 4:**
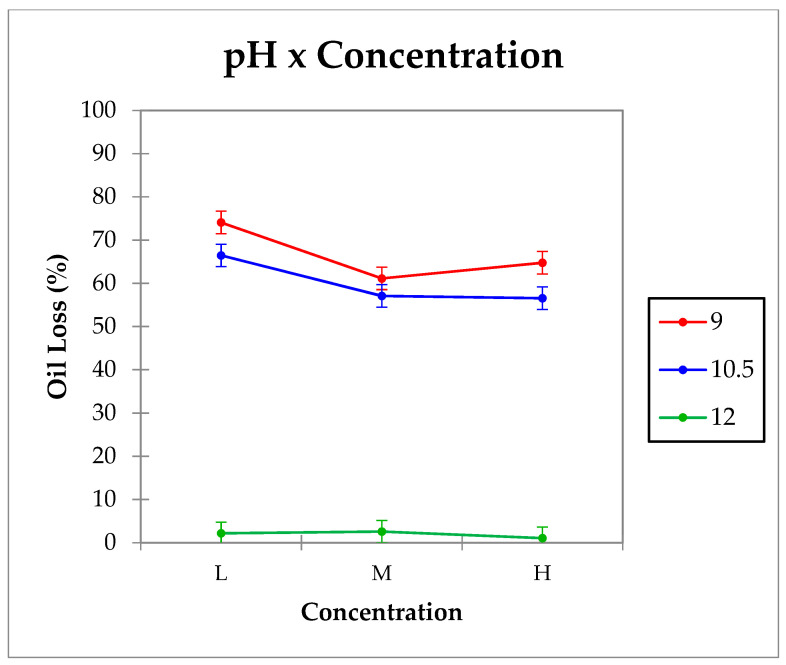
Emulsions oil loss interaction graph.

Physical stability was investigated by determining the oil lost under centrifugal forces (oil loss %). Interactions (*p <* 0.05) between the by-product’s concentration and the pH of the aqueous phase for oil loss values were observed and shown in Figure 4. In emulsions with pH 9 and 10.5, oil loss values were higher than 50% regardless of the by-product’s concentration, being significantly (*p <* 0.05) higher in emulsions with low concentration. No significant differences (*p >* 0.05) were observed among emulsions produced with medium and high by-product concentration. However, samples with high pH (pH 12) were the most stable and showed no significant differences (*p >* 0.05) among them. These results indicate that pH and concentration have an influence on HIPE gels stability; increasing pH is the most important factor. HIPE gels with pH 12 were the most stable.

**Figure 5:**
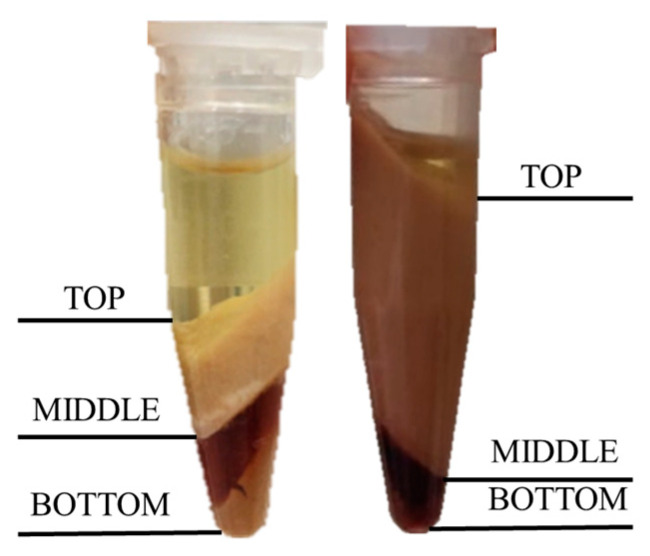
Appearance of the emulsions after centrifugation. (**Left**) sample 9H; (**right**) sample 12H. After centrifugation, three layers appeared and were identified.

After centrifugation, three layers were observed in low stability emulsions, shown in Figure 5. The top layer corresponded to oil, middle layer to the emulsion, and bottom layer to the aqueous phase. Increasing the pH of the medium increased the stability; this has been attributed by other authors to an increase in the charge density of the proteins which produced higher repulsions among droplets as discussed by Vélez-Erazo et al. [36].

Stabilizing thermodynamically unstable systems with raw by-products is difficult; therefore, diluted emulsions are usually produced to evaluate their properties. Oil-in-water emulsions (50/50 (*wt*/*wt*)) were prepared by Huc-Mathis et al. [31], using plant powders (apple pomace and oat bran) and showed similar G′ and G″ values to the 9L sample in this study. Joseph et al. [37] also fabricated 10/90 and 50/50 (*wt*/*wt*) emulsions using cocoa, rapeseed, and lupin hull powders but no rheological measurements were conducted, although relatively long-term (14–70 days) stable emulsions were produced depending on the plant material used for stabilization. The trend when working with by-products is to produce Pickering emulsions due to the heterogeneous composition of the raw materials consisting of soluble and insoluble compounds.

### 2.5. Color Analysis

Table 2 shows the color parameters L*, C*, and h*. There were no interactions (*p >* 0.05) between the by-product’s concentration and pH of the aqueous phase for the studied parameters, but both factors had a significant effect (*p <* 0.05) on these parameters.

Luminosity (L*) was mainly influenced by the by-product concentration. All samples had L* values ranging from 45 to 60, thus, all showed a medium luminosity (0 = black; 100 = white). Regarding the influence of the pH of the aqueous phase, only significant differences (*p <* 0.05) were found between samples 9H and 12H, being L* higher at higher pH. When comparing emulsions with the same pH, samples with a higher concentration (9H and 12H) had inferior L* values (*p <* 0.05) than those with low concentration (9L and 12L). No significant differences (*p >* 0.05) were found among emulsions with low and medium by-product concentration for the same pH value.

The chroma (C*) parameter had a similar but inverse behavior than lightness because chroma only depends on the light removed by scattering, whereas the lightness also is influenced by the direction where the light is scattered [38]. When the effect of the pH of the aqueous phase was assessed for emulsions with the same by-product concentration, significant differences (*p <* 0.05) were observed among samples with low (9L and 12L) and high concentration (9H and 12H). C* value increased (*p <* 0.05) when increasing pH, except for the medium by-product concentrations. When comparing emulsions with the same pH, at low and medium pH there were differences (*p <* 0.05) between samples with low and high concentration, being C* higher in emulsions with high concentration. No differences were found (*p >* 0.05) among samples with medium and high by-product concentration, except for pH 12 where significant differences (*p <* 0.05) among the three emulsions were detected, increasing C* when increasing by-product’s concentration. Generally, C* values increased both as the by-product’s concentration and the pH of the aqueous phase increased. Umaña et al. [39] prepared oil-in-water emulsions using a concentrate of mushroom by-products and indicated that increasing the concentration of the concentrate produced a decrease in L* and an increase in a* and b*, thus C* increased.

Finally, for most emulsions, the hue angle (h*) value ranged from 70° to 90° (Table 2). Only sample 9L had a higher value than 90°. This emulsion had a yellow-greenish color. However, the rest of the samples were in the red-yellow range, so most emulsions presented orange tones. Increasing the pH of the aqueous phase of the emulsion showed more reddish emulsions. In terms of by-product concentration, when comparing emulsions with the same pH, the increase in by-product’s concentration also generated more reddish emulsions. However, no significant differences were found (*p <* 0.05) between the 9M and 9H emulsions, nor between the 10.5M and 10.5H emulsions. All the emulsions formulated at pH 12 showed significant differences (*p <* 0.05) between them, being those made with a lower concentration of by-product (12L) present significantly (*p <* 0.05) higher values of h*. The influence of by-product concentration on the hue angle appeared to be greater at high aqueous solution pH values. The reason behind the diminution of h when increasing the pH, making samples more orange, might be due to the effect of the pH on hop particles. According to Verzele et al. [40], spent hops are a rich source of xanthohumol. An increase in the medium’s pH favors the separation of this flavonoid from the cell wall. This compound, when in solution, exhibits a deep red color, which might explain the change in the hue angle to reddish tones.

## 3. Conclusions

The increase in by-product concentration and the pH of the aqueous phase allowed development of HIPE gels with structural and physical stability, with homogeneously distributed oil droplets of regular size and polyhedral structure.

Rheology data proved a more stable structure at higher pH and higher by-product concentration due to the higher amount of yeast cell debris and the cleavage of the yeast cell wall that releases intracellular compounds. This release increased the viscosity of the aqueous phase and mannoprotein’s solubility. Luminosity decreased when the by-product’s concentration increased. Increasing by-product concentration and the pH of the aqueous phase increased chroma values and diminished hue value angle, favoring the development of orange-colored emulsions.

This study widens the possibilities to valorize the by-products from the modern brewing industry as stabilizers when designing emulsions. However, further investigation should be conducted to expand the knowledge of these by-products for designing HIPE gels for its implementation on an industrial scale.

## 4. Materials and Methods

### 4.1. Materials

The by-product mixture of trub and spent brewer yeast was kindly supplied by TYRIS Craft & Creative Beers (Valencia, Spain). Refined sunflower oil (Hacendado, Valencia, Spain) was acquired from a supermarket and distilled water was used throughout this study.

### 4.2. Methods

#### 4.2.1. By-Products’ Powder Preparation

The mixture of trub and spent yeast was stored at −8 °C immediately after collection from its production tank to prevent microbial growth. The sample was defrosted and centrifuged (Sorvall Super T21, GMI, Ramsey, NJ, USA) at 17,000× *g* for 10 min at 4 °C to remove residual beer liquor. Then, it was vacuum dried (Vaciotem-T, J.P. SELECTA, Barcelona, Spain) at 40 °C and −0.7 bar until reaching constant weight. After that, the mixture was milled using a thermostatic grinder (IKA M20, Staufen, Germany) to reduce the powder’s size before ball milled.

The water content of the powder was determined after vacuum drying (Vaciotem-T) at 60 °C for 24 h. Particle size was determined using a vibratory sieve shaker with automatic amplitude control (RP 200N SILENT, CISA, Barcelona, Spain). Color attributes (CIEL*a*b*, standard light source D65 and standard observer 10°) were measured with a spectrophotocolorimeter Minolta CR-400 (Konica Minolta Sensing, Inc., Osaka, Japan).

#### 4.2.2. Dispersion and High Internal Phase Emulsion Gels Preparation

Powder was dispersed in water and stirred for 1 h to ensure complete dissolution. Then, pH was set by carefully adding 6 M NaOH. After, the aqueous phase was stirred with a magnetic stirrer for 2 h at room temperature (20–25 °C). The homogenization process was conducted using an Ultra-Turrax (Ultraturrax T18, IKA, Staufen, Germany) with a rotor dispersor of 12.7 mm, based on the procedure described by Wijaya et al. [41] with slight modifications. Briefly, 5 g of the aqueous phase was dispersed for 30 s at 8000 rpm, then 20 g of oil was slowly added to the water phase at 14,000 rpm. Once all the oil phase was added, the homogenization process ended with 30 s at 16,000 rpm. The self-standing emulsions were stored at 4 °C before analysis on the following day.

According to pH and concentration, nine samples were evaluated and named, as shown in Table 3. All analyzes were carried out in triplicate.

#### 4.2.3. Microstructure

The microstructure was studied using a Cryo-field emission scanning electron microscopy. The samples were frozen by immersion in slush nitrogen and after being fractured, etched, and coated with platinum, they were observed at 15 kV at a working distance of 15 mm in a microscope Ultra55 FESEM (Zeiss, Oberkochen, Germany).

#### 4.2.4. Rheological Behavior

A rotational rheometer (Kinexus Pro+, Malvern Panalytical, Worcestershire, UK), equipped with a Peltier plate cartridge was used to perform tests at 5 °C [41,42]. A 40 mm diameter plate–plate sensor geometry and 1 mm gap were used. Samples were left for 3 min to rest after reaching the desired gap. Amplitude sweeps (0.01–100 Pa, 1 Hz), frequency sweeps (0.1–10 Hz, 1 Pa), and flow curves (0.1–100 s^−1^) were conducted.

#### 4.2.5. Physical Stability

Determination was made by measuring the oil lost under centrifugal forces according to the method of Vélez-Erazo et al. [35] with minor modifications. Briefly, approximately 1 g of emulsion was inserted in a 1.5 mL Eppendorf tube and centrifuged at 11,200× *g* for 30 min at 4 °C. Free oil was carefully removed with a Pasteur pipette. Results (expressed as g of oil loss per 100 g of sample) were calculated using Equation (1), where m_i_ refers to the weight of the tube with emulsion, m_f_ refers to the tube with emulsion after centrifugation and free oil removal, and m refers to the weight of the Eppendorf tube.

(1) $$\mathrm{Oil}\;\mathrm{Loss}\;\left(\%\right)=\frac{m_{i}-m_{f}}{m_{i}-m}\times 100$$

#### 4.2.6. Color Analysis

For color determination of emulsions, a Minolta CM-3600d spectrocolorimeter (Minolta Co., Tokyo, Japan) was used. Emulsions were placed over 40 mm × 12 mm soda-lime glass Petri dishes and covered, ensuring no appearance of bubbles. Color was measured using the CIEL*a*b* color coordinates considering the standard light source D65 and the standard observer 10°. In this system, L* indicates the level of lightness/darkness, C* is related to the purity of the color, and the hue angle (h*) shows the tonality of the color.

#### 4.2.7. Statistical Analysis

A categorical multifactorial experimental design with two factors—pH of the aqueous phase and concentration of the by-product—was used to characterize all samples. Analysis of variance (ANOVA) was performed on the data. The least significant differences (Fisher’s LSD test) with a 95% confidence were used to compare the obtained mean values (*p <* 0.05).

All the data were analyzed using XLSTAT 2018.1 (Addinsoft, Barcelona, Spain).

## Figures and Tables

**Table 1:** Rheological parameters of the emulsions.

Sample	G′ (Pa)	G″ (Pa)	Viscosity (Pa·s)
9L	n.d.	n.d.	n.d.
9M	206 (12) ^ab^	43 (2) ^ab^	0.24 (0.04) ^a^
9H	261 (19) ^bc^	67 (4) ^bc^	0.56 (0.02) ^c^
10.5L	69 (9) ^a^	19 (2) ^ab^	0.24 (0.03) ^a^
10.5M	391 (49) ^cd^	80 (11) ^c^	0.67 (0.04) ^d^
10.5H	670 (88) ^e^	160 (16) ^e^	0.55 (0.03) ^c^
12L	78 (6) ^a^	29 (3) ^a^	0.41 (0.03) ^b^
12M	515 (30) ^d^	129 (6) ^d^	1.51 (0.06) ^e^
12H	1228 (37) ^f^	248 (41) ^f^	2.61 (0.03) ^f^

G′ and G″ are determined at 1 Hz and 1 Pa, and viscosity at 50 s^−1^. Different superscript letters within the same column mean significant differences between samples (*p <* 0.05).

**Table 2:** Color parameters of the emulsions.

Sample	L*	C*	h*
9L	55.77 (0.73) ^cd^	18.50 (0.96) ^a^	93.68 (1.14) ^f^
9M	54.19 (1.32) ^bcd^	21.66 (1.24) ^cd^	89.16 (0.52) ^e^
9H	47.74 (0.93) ^a^	21.04 (0.58) ^bc^	86.91 (0.81) ^e^
10.5L	57.66 (0.71) ^d^	19.80 (0.93) ^ab^	87.29 (1.10) ^e^
10.5M	53.77 (0.77) ^bcd^	21.93 (0.08) ^cd^	82.27 (1.51) ^d^
10.5H	50.91 (2.86) ^ab^	22.73 (0.89) ^d^	83.48 (1.55) ^d^
12L	56.79 (5.08) ^d^	20.61 (0.03) ^bc^	77.95 (1.55) ^c^
12M	56.65 (3.28) ^d^	22.97 (0.44) ^d^	71.37 (0.50) ^a^
12H	52.49 (1.52) ^bc^	24.51 (1.29) ^e^	74.67 (0.27) ^b^

Different superscript letters within the same column mean significant differences between samples (*p <* 0.05).

**Table 3:** Naming and composition of the samples according to the pH of the aqueous phase and the concentration of by-product in emulsions.

	pH 9			pH 10.5			pH 12		
	9L	9M	9H	10.5L	10.5M	10.5H	12L	12M	12H
By-product’s concentration (%)	1	2.5	4	1	2.5	4	1	2.5	4
Sunflower oil (%)	80	80	80	80	80	80	80	80	80
Water (%)	19	17.5	16	19	17.5	16	19	17.5	16

## Data Availability

Research data are not shared.
